# Zolpidem-induced sneezing: A case report of positive rechallenge

**DOI:** 10.1097/MD.0000000000009918

**Published:** 2018-03-02

**Authors:** Razie Amraei, Abdolhamid Parsa, Mohammad Babaeian

**Affiliations:** Shahid Behshti University of Medical Sciences, Tehran, Iran.

**Keywords:** insomnia, rechallenge, sleep, sneezing, zolpidem

## Abstract

**Introduction:**

Zolpidem, as an imidazopyridine, is a widely prescribed drug in clinical practice for short-term treatment of insomnia. Nevertheless, there have been a number of cases associated with the adverse effects of the stated drug recently. Further to the existing reports of adverse reactions to zolpidem, the current script is going to report a case in which zolpidem has induced acute repetitive sneezes.

**Conclusions:**

A high dose of zolpidem may contribute to interruption to the neurons function involved in the sneezing pathway.

## 1 Introduction

Zolpidem (ZLP) as an imidazopyridine is a popular hypnotic medicine indicated for short-term treatment of insomnia.^[1]^ Although ZLP is regarded safer than benzodiazepines, its side effects are common and cannot be neglected and several systemic descriptive studies have been undertaken.^[2,3]^ The most common reported side effects were nausea, dizziness, malaise, hallucination, nightmares, agitation, and headache.^[4]^ In addition, several case reports of abuse, dependence, and withdrawal syndrome have been published.^[5,6]^ It is valuable to state that in the majority of these cases, the recommended dose of ZLP was exceeded. ^[2,6]^

Further to the accumulating reports of ADRs (adverse drug reaction)s related to ZLP use, we present a case in which a high dose of ZLP has induced consecutive sneezing.

## 2 Case presentation

An unmarried 29-year-old male who was living alone in a microapartment in Tehran, Iran, without any disease or respiratory disorder, and with a history of seasonal rhinitis occurring in the spring, came to a community pharmacy in December 2016 to buy antihistamine to stop sneezing.

He declared that he had been using ZLP as a sleeping pill for a while, however in the aftermath of frequent use; he had become interested in the agreeable mental experiences of using ZLP. The most powerful inductors of his repetitive abuse of ZLP include an overwhelming increase in feeling depression and loneliness during nights, as he said. He had a history of Pro re nata use of ZLP during the past years. After a while, not only ZLP was not able to put him to sleep any longer, but it also led him to spend time doing his desirable things such as cooking, tidying up, surfing the internet, chatting up with friends, listening to music, making phone calls, and watching movies.

The prevalent unfavorable effects of using ZLP, as described by him, were nausea, vomiting, acute diarrhea (upon taking the pills), constipation (in the case of frequent use), dyspepsia, stomach cramps, abdominal pain, nightmares, dizziness, hangover, blurred vision, visual hallucination, headache, imbalance, fatigue, feeling a bitter taste in saliva, and feeling a chlorine/ammonia type odor while breathing through nose deeply.

One night in, as a groove, after a raid of gloomy and negative thoughts, with a great desire for drug abusing, he took 6 pills (60 mg) of ZLP with a full glass of water. After a few minutes, he experienced vigorous and uncontrollable sneezes happening every 20 seconds for about 30 minutes. The unprecedented side effect got disappeared in about 1 hour completely but was occurring nearly every 1 hour in the next morning. He performed rechallenge for 5 consecutive nights, by using 60 mg of ZLP each time, staying awake and observing the changes. He believed that all of the mentioned adverse effects including sneezing had repeated for each time.

Both cases tended to complete the informed consent, voluntarily. The study involves patient consent. And all the data are confidential.

## 3 Discussion and conclusion

Sneezing or sternutation is a primitive neuromuscular physiological response to irritation.^[7,8]^ Sneezing can be due to allergy to triggers, such as dust, air pollutants, dry air, spicy foods, certain medicines, or powders, strong emotions, breathing in corticosteroids, common cold, the flu or drug withdrawal.^[9]^ Also, sneezing during the insertion of a peribulbar block under sedation and following exposure to sun light has been reported.^[8,10]^

Sneezing is a rarely concerned symptom in neurological practice. In cats, sneeze-evoking center is located in the medulla.^[11–13]^ Seijo-Martínez et al^[14]^ have reported a case with abnormal sneezing secondary to an infarction in the right latero-medullary region. Fink^[15]^ has demonstrated the anticipated sneeze-evoking center in the rostral dorsolateral medulla in a 23-year-old female.

The sneezing reflex follows a very complex pathway requiring harmonic communications between the nerves terminals and trigeminal afferent impulses as well as from glottic and aspiratory muscles in the efferent limb. When the reflex pathway is distracted, sneezing may repeat and get more vigorous insofar as some cases become even disabled.^[7,11]^

ZLP is a drug with selective hypnotic effect on type A gamma-aminobutyric acid (GABA_A_) receptors. It has a good bioavailability, short onset of action, and well tolerated.^[16–18]^ Despite the stated benefits, there have been various notable adverse effects reports of ZLP so far, and a variety of other rare side effects are being reported due to widespread use of ZLP in clinical practice.^[18,19]^ ZLP is potent for both medical misuse and recreational use to get a “high.”^[19–21]^

Niddam et al ^[22]^ have reported notable levels of [^3^H] ZLP binding sites in the brain medulla. The medulla oblongata helps regulate breathing, heart and blood vessel function, digestion, sneezing, and swallowing.^[23–25]^

According to the patient's remarks based on Naranjo algorithm,^[26]^ the case takes score 5 and we may say that the patient has experienced a “probable ADR” related to ZLP.

In the light of the above-mentioned points, the high dose of ZLP taken by the case might have contributed to interruption to the neurons function involved in the sneezing pathway.

To the best of our knowledge, this is the first reported case of sneezing upon taking ZLP tablets. As underlined by FDA^[27]^ and as a suggestion, it is recommended to use ZLP at the lowest effective dose for very least period of time as necessarily needed.

As this case is based on 1 personal declaration and due to possible contribution of other factors such as allergy, diet, or gender, supplementary investigations are needed to confirm or refute this rare side effect of ZLP as a widely used drug in the world and Iran.^[28]^

## Acknowledgment

We would like to acknowledge Mr Majid Ghobadi for his tremendous help and valuable suggestions.
