# Zolpidem-induced sneezing: A case report of positive rechallenge: Erratum

**DOI:** 10.1097/MD.0000000000010749

**Published:** 2018-05-11

In the article, “Zolpidem-induced sneezing: A case report of positive rechallenge”,^[1]^ which appeared in Volume 97, Issue 9 of *Medicine*, the second sentence of the first paragraph should appear as “Although ZLP is regarded safer than benzodiazepines, its side effects are common and cannot be neglected and several systematic descriptive studies have been undertaken.^[2,3]^”
